# A Synthetic Uric Acid Analog Accelerates Cutaneous Wound Healing in Mice

**DOI:** 10.1371/journal.pone.0010044

**Published:** 2010-04-06

**Authors:** Srinivasulu Chigurupati, Mohamed R. Mughal, Sic L. Chan, Thiruma V. Arumugam, Akanksha Baharani, Sung-Chun Tang, Qian-Sheng Yu, Harold W. Holloway, Ross Wheeler, Suresh Poosala, Nigel H. Greig, Mark P. Mattson

**Affiliations:** 1 Laboratory of Neurosciences, National Institute on Aging Intramural Research Program, Baltimore, Maryland, United States of America; 2 Research Resources Branch, National Institute on Aging Intramural Research Program, Baltimore, Maryland, United States of America; 3 Biomolecular Science, University of Central Florida, Orlando, Florida, United States of America; 4 Department of Pathology and Medical Education, University of Central Florida, Orlando, Florida, United States of America; 5 Department of Neuroscience, Johns Hopkins University School of Medicine, Baltimore, Maryland, United States of America; Brunel University, United Kingdom

## Abstract

Wound healing is a complex process involving intrinsic dermal and epidermal cells, and infiltrating macrophages and leukocytes. Excessive oxidative stress and associated inflammatory processes can impair wound healing, and antioxidants have been reported to improve wound healing in animal models and human subjects. Uric acid (UA) is an efficient free radical scavenger, but has a very low solubility and poor tissue penetrability. We recently developed novel UA analogs with increased solubility and excellent free radical-scavenging properties and demonstrated their ability to protect neural cells against oxidative damage. Here we show that the uric acid analog (6, 8 dithio-UA, but not equimolar concentrations of UA or 1, 7 dimethyl-UA) modified the behaviors of cultured vascular endothelial cells, keratinocytes and fibroblasts in ways consistent with enhancement of the wound healing functions of all three cell types. We further show that 6, 8 dithio-UA significantly accelerates the wound healing process when applied topically (once daily) to full-thickness wounds in mice. Levels of Cu/Zn superoxide dismutase were increased in wound tissue from mice treated with 6, 8 dithio-UA compared to vehicle-treated mice, suggesting that the UA analog enhances endogenous cellular antioxidant defenses. These results support an adverse role for oxidative stress in wound healing and tissue repair, and provide a rationale for the development of UA analogs in the treatment of wounds and for modulation of angiogenesis in other pathological conditions.

## Introduction

The rapid and coordinated responses of several different cell types, including circulating platelets and immune cells, and intrinsic keratinocytes, fibroblasts and vascular endothelial cells, are required for the proper healing of full-thickness wounds [1], [2]. Within seconds to minutes of the injury, platelets are recruited to the wound site to aid in clot formation, and inflammatory processes mediated by innate molecular cascades and infiltrating leukocytes occur. These rapid responses limit blood loss and guard against infectious agents. During the ensuing days keratinocytes migrate over the injured dermis and the granulation tissue and proliferate to restore the barrier function of the skin. Concomitantly, fibroblasts migrate into the clot and proliferate and angiogenesis occurs at the wound edge. Then, in a slower process that occurs over a period of months to several years, tissue remodeling occurs and (in mammals) a scar is formed by collagen-producing fibroblasts.

The cellular signaling mechanisms that regulate wound healing are complex and poorly understood, but recent findings suggest key roles for growth factors such as EGF, FGF2 and HGF, the cytokine TGFβ and the cell fate regulator Notch [2]–[7]. From a clinical perspective, an attractive feature of full-thickness wounds is that treatments can be applied topically, thus reducing or eliminating adverse effects on other organs. Thus, topical application of ligands for several growth factors have been reported to enhance wound healing in animal models [3], [4], [7] and, in some cases, in human subjects [8], [9].

A rapid increase in oxygen free radical production and oxidative damage to proteins, DNA and lipid occurs in cells within and adjacent to the wound site [10]–[12]. Some reactive oxygen species appear to serve beneficial signaling roles in the recruitment of immune cells and clearance of cellular debris, for example [13]. However, oxidative stress is detrimental to multiple cellular processes that occur during the period of tissue healing and remodeling that occurs over a period of days to weeks after the injury [14]–[16]. Reactive oxygen species (ROS) generated in wounded dermal cells include superoxide, hydrogen peroxide, hydroxyl radical (formed by the interaction of hydrogen peroxide with Fe^2+^) and peroxynitrite (formed by the interaction of superoxide with nitric oxide) [17]. These ROS result in membrane lipid peroxidation, protein oxidation and damage to nucleic acids, any of which may impair cellular processes involved in wound healing including proliferation and migration of epidermal cells, and angiogenesis [18]. The increased ROS levels experienced by cells in wounded tissue may be exacerbated by the depletion of antioxidant enzymes including Cu/Zn superoxide dismutase (SOD1) and glutathione peroxidase [19].

Uric acid (UA) is perhaps best known for its central role in gout, a disorder characterized by elevated levels of UA resulting in its precipitation to form crystals that are deposited in joint tissues where they cause inflammation and pain [20]. On the other hand, low levels of uric acid are associated with several major disorders including Alzheimer's and Parkinson's diseases, and multiple sclerosis [21]. Soluble UA functions as a free radical scavenger of hydroxyl radical and peroxynitrite and, in fact, UA is the most prominent antioxidant in the blood of humans and birds [22], [23]. Previous findings have demonstrated a benefit of intraperitoneal or intravenous administration of UA in experimental models of several disorders that involve increased oxidative stress including multiple sclerosis [24], Alzheimer's disease [25], stroke [26] and spinal cord injury [27]. However, the relative insolubility of UA and its ability to form toxic crystals reduces its clinical utility. We recently reported on the development of novel UA analogs with greatly increased solubility and potent antioxidant acitivity [28]. In vitro and cell culture screening identified 1, 7-dimethyluric acid and 6, 8-dithiouric acid as two analogs with high antioxidant and neuroprotective activities. When administered intravenously in mice, both UA analogs lessened damage to the brain and improved functional outcome in an ischemia–reperfusion mouse model of stroke [28]. In the present study we provide evidence that topical administration of 6, 8-dithiouric acid accelerates wound healing in mice by a mechanism that may involve actions of the UA analog on fibroblasts, keratinocytes and vascular endothelial cells. These findings show that soluble UA analogs can improve wound healing and suggest novel therapeutic uses for UA analogs in clinical settings.

## Results

### 6, 8 dithio-uric acid enhances the motility and proliferation of vascular endothelial cells

**Figure 1:**
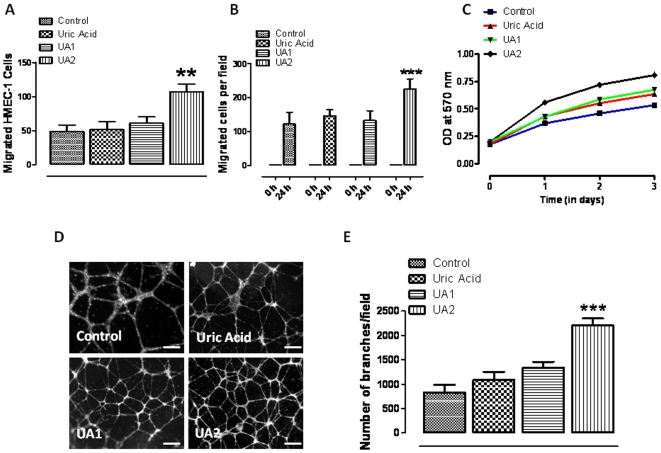
A uric acid analog enhances the motility and proliferation of human vascular endothelial cells. **A.** Cultured HMEC-1 were treated with vehicle (control), uric acid, 1, 7 dimethyl-uric acid (UA1) or 6, 8 dithio-uric acid (UA2) (15 µM) in conditioned medium and plated into chemo-attractant medium consisting of regular growth medium supplemented with 10% fetal bovine serum and other growth factors, and cell migration was evaluated using a 24 well Transwell chamber assay. (values are the mean and SEM for cells per 100×field; n = 3–4). **p,0.01 compared to control values. **B.** HMEC-1 monolayers were mechanically wounded with a sterile tip of 20–200 ml pipette tip following treatment without (control) or with UA, UA1 or UA2 (15 µM). Values are the mean and SEM (n = 3 separate experiments). *p,0.001. **C.** Cultured HMEC-1 were treated with vehicle, UA, UA1 or UA2 (15 µM) for the indicated time relative cell numbers were quantified by O.D readings (n = 4–6 experiments), *p<0.01. **D&E.** HMEC-1 cells were seeded on Matrigel-precoated wells and cultured in the presence of low-serum medium with vehicle (control), UA, UA1 or UA2 (15 µM). Tube formation, designated as the number of branch points/100X field) was evaluated 18 h after cell plating. Representative images are shown in D and quantitative data in E. Values are the mean and SEM (n = 12–16 cultures). *p,0.05; *p,0.001. Scale bars represent 100 µm.

By producing microvessels that provide nutrients and oxygen to growing dermal cells, angiogenesis plays a critical role in wound healing [29]. We therefore determined whether UA analogs affect angiogenic behaviors of cultured vascular endothelial cells. Human microvascular endothelial cells (HMEC-1 cells) were treated with vehicle (control), uric acid, 1, 7 dimethyl-uric acid (UA1) or 6, 8 dithio-uric acid (UA2) and cell migration was evaluated using a 24 well Transwell chamber chemoatraction assay. The concentration of UA and UA analogs used (15 µM) was chosen based on our previous studies [28] and preliminary dose-findings experiments. UA2, but not UA or UA1, significantly enhance vascular endothelial cell migration rate toward the chemoatractant medium (Fig. 1A). We next employed a scrape wound assay in which monolayers of cultured vascular endothelial cells were mechanically wounded with a pipette tip. The migration of cells across the substrate in the wound chasm was significantly enhanced in cells treated with UA2 compared to those treated with vehicle, UA or UA1 (Fig. 1B). The proliferation rate of the endothelial cells during a 3 day period was significantly greater in endothelial cells treated with UA2 than controls or cells treated with UA or UA1 (Fig. 1C). These results suggest that UA2 can enhance two behaviors of endothelial cells, proliferation and directed cell migration, that are critical for angiogenesis in wound healing. Blood vessel formation requires that endothelial cells interact with each other to form tubes [30]. We found that the ability of endothelial cells to form three dimensional tubes when grown in matrigel was significantly increased by more than two-fold in the presence of U2 (Fig. 1D, E). In contrast, UA had no significant effect on endothelial cell tube formation and UA1 increased tube formation by only 25%.

### 6, 8 dithio-uric acid enhances the motility and proliferation of fibroblasts and keratinocytes

**Figure 2:**
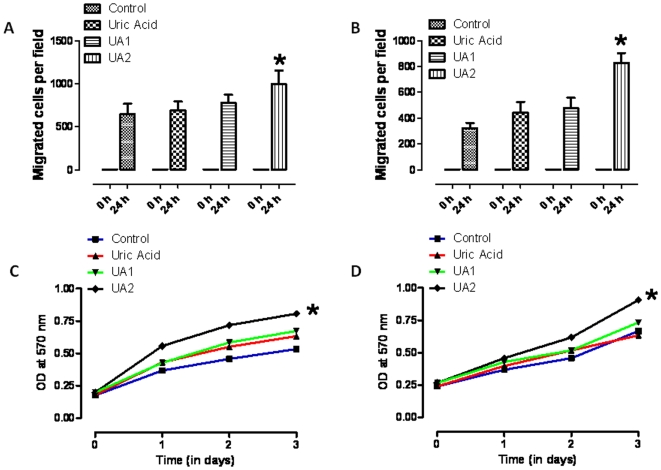
A uric acid analog enhances the motility and proliferation of keratinocytes and fibroblasts. A–D. Monolayers of cultured keratinocytes (A) or fibroblasts (B) were treated with vehicle (Control), UA, UA1 or UA2 (15 µM) and were then subjected to scratch wounding. Eighteen hours after wounding, images of the wound area were acquired and the number of cells per field that had migrated into the cell-free wound zone was determined for each culture. Quantitative data A&B are shown. Values are the mean and SEM (n = 3 separate experiments). *p,0.05. C and D. Cultured keratinocytes (C) and fibroblasts (D) were treated with vehicle (Control), UA, UA1 or UA2 (15 µM) for the indicated time periods and relative cell numbers were estimated by O.D readings, p<0.01. Values are the mean and SEM (n = 4–6 experiments).

The reformation of a functional germ and toxin-resistant dermis and epidermis in a wound requires the proliferation and migration of both fibroblasts and keratinocytes [31]. We first evaluated the migration of keratinocytes and skin fibroblasts using the cell monolayer/scratch wound assay. Treatment with UA2, but not UA or UA2, significantly increased the rate of migration of both keratinocytes and fibroblasts into the wound area compared to the migration rates of these cells in control cultures (Fig. 2A, B). We also found that U2 enhanced the proliferation of keratinocytes and fibroblasts, whereas UA and UA1 did not affect cell proliferation significantly (Fig. 2C, D).

### 6, 8 dithio-uric acid accelerates the healing of full-thickness wounds in mice

**Figure 3:**
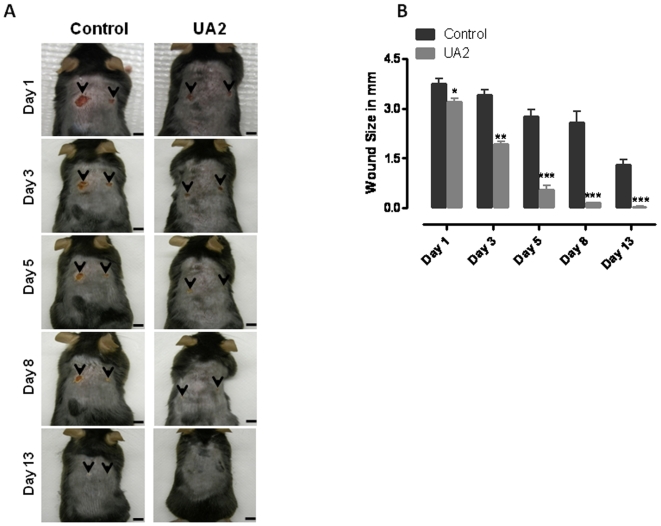
Topical application of 6, 8 dithio-uric acid accelerates the healing of full-thickness wounds in mice. Two full-thickness wounds were induced in vehicle- and UA2-treated (100 µM solution) mice. A. Images of a representative mouse from each group taken on post-injury days 1, 3, 5, 8 and 13 are shown. B. Wound sizes at the indicated time points in Control and UA2 (15 µM) topical treated mice. Values are the mean and SEM (n = 6 mice per group). ***p<0.001, **p<0.01, *p<0.05 #p<0.01 compared to the control value. Scale bar = 4 mm.

Because UA2, but not UA or UA1, affected the behaviors of cultured fibroblasts, keratinocytes and vascular endothelial cells in ways that would be expected to enhance wound healing in vivo. We therefore employed a mouse model to determine whether topical application of UA2 would modify the healing of full-thickness dermal wounds. Two full-thickness wounds were induced in young adult male C57BL/6 mice and then UA2 or vehicle was applied topically to the wounds once daily. Images of the wounds were acquired on post-injury days 1, 3, 5, 8 and 13 and wound sizes were quantified. On post-injury day 1 the size of wounds in UA2-treated mice was approximately 15% smaller than wound size in control mice (Fig. 3A, B). Subsequently, there was a rapid acceleration of wound healing in the UA2-treated mice such that on days 3 and 5 the wounds were approximately 50% and 80% smaller than controls, respectively. By day 8 the wounds of UA2-treated mice were completely closed, whereas the wounds of control mice had not yet healed completely (Fig. 3B). We observed no adverse effects of topical UA2 treatment on the body weight, general health or behavior of the mice.

**Figure 4:**
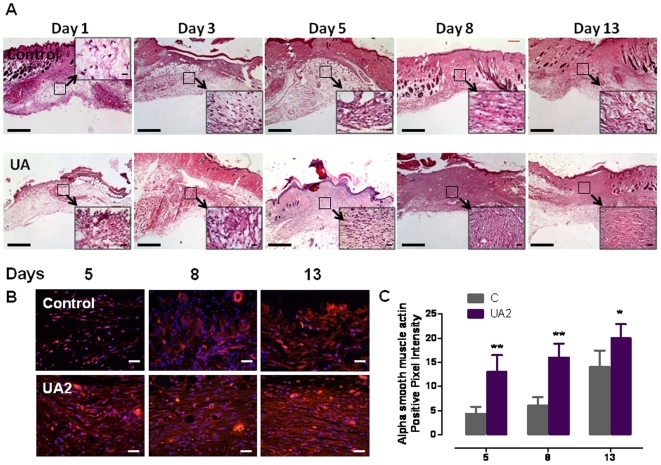
Histological features of wound healing in mice treated with UA2 or vehicle. **A.** Images of skin tissue sections stained with hematoxylin and eosin showing histological changes during the wound healing process in control mice, with uric acid analog at post-injury days 1, 3, 5, 8 and 13. UA2 treated mice exhibited enhanced restoration of dermal and epidermal tissues in the wound. See File S1 for a detailed description of histological changes in the different groups of mice. Scale bar = 1 mm. These images are representative of 12 wounds in 6 mice for each treatment group. **B.** Immunostaining for α smooth muscle actin showing wound healing tissues on days 5, 8 and 13 in mice treated with UA2 or vehicle. Pictures showing enhanced myofibroblast differentiation in UA2 treated groups compared to controls in days 5, 8 (**p<0.01) and 13 ((*p<0.05). Scale bar = 25 µm. **C.** Quantification of alpha smooth muscle actin positive staining.

**Figure 5:**
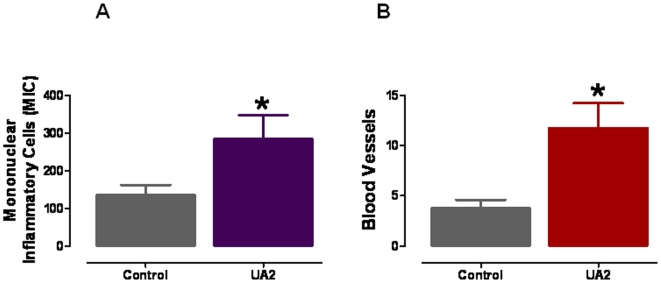
Wounds treated with a uric acid analog exhibit enhanced infiltration of immune cells and enhanced growth of blood vessels. **A.** Numbers of mononuclear immune cells in wound tissue 5 days after the injury in control and UA2-treated mice. **B.** Numbers of blood vessels in wound tissue 5 days after the injury in control and UA2-treated mice. Values are the mean and SEM (n = 6 mice). *p<0.05.

In a parallel experiment, we euthanized mice in UA2-treated and control groups at post-injury days 1, 3, 5, 8 and 13 and then performed a histological evaluation of skin tissue sections stained with hematoxylin and eosin. UA-treated mice exhibited enhanced restoration of dermal and epidermal tissues in the wound (Figs. 4, 5 and see File S1 for a detailed description of histological changes in the different groups of mice). Examination of the skin tissue sections revealed that, in addition to accelerating the closure of the wounds, UA2 treatment resulted in restoration of a near-normal dermis and epidermis by post-injury days 8 and 13 (Figs. 4, 5). In contrast, the skin tissue in the closed wounds of control mice (post-injury day 13) had not been restored and exhibited acellularity, vacuolation and accumulations of cell debris. Based on our histological evaluation it is clear that at days 8 and 13 denser and extended granulation tissue is seen in the UA2-treated group compared to the control group. While this is clearly beneficial for the healing process, it remains to be seen whether this may also result in temporary hypertrophic scar formation. Since enhanced myofibroblast differentiation may explain the acceleration of wound closure in UA2-treated mice, we stained wound tissues at post injury days 5, 8 and 13 with an antibody against alpha smooth muscle actin, a differentiation marker of smooth muscle cells (Fig. 4B, C). The results revealed that enhanced myofibroblast differentiation occurred in the UA2-treated wounds. Histomorphometric analysis (Fig. 5A, B) showed that UA2-treated wounds on day 5 had more infiltrated mononuclear inflammatory cells and new blood vessels compared to vehicle-treated wounds (Fig. 5A, B).

### UA2 treatment results in increased levels of SOD1, and decreased accumulation of protein carbonyls and nitrated proteins, in the wound tissue

**Figure 6:**
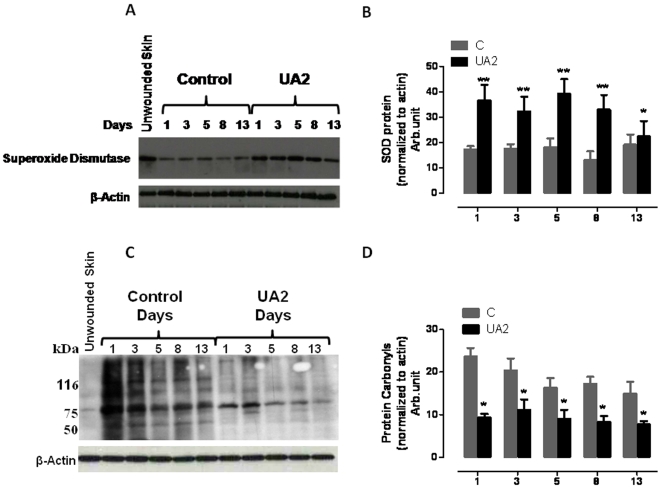
Levels of Cu/Zn superoxide dismutase (SOD1) are increased in wound tissue from mice treated with UA2 (n = 4). **A.** Mice were treated with vehicle (control) or UA2 for the indicated number of days post-injury along with unwounded skin tissue. Wound tissue samples were then removed and were subjected to immunoblot analysis (40 µg protein/lane) using antibodies to SOD1, and actin (44 kDa). **B.** Densitometric analysis of band intensity (Image J, NIH) of immunoblots showed a significantly increased level of SOD in the skin tissue at 1, 3, 5, 8, 13 days in UA2 treated mice compared to control mice. * P<0.01 compared to values for each of the other groups. Statistical comparisons were made using ANOVA with Newman-Keuls post hoc tests for pair wise comparisons using GraphPad Prism version 5.00 for Windows, GraphPad Software, San Diego California USA, www.graphpad.com. **C.** Enhanced oxidative stress in control wound tissues compared to UA2 treated at days 1,3,5,8 and 13. Lysates (20 µg of total protein) were analyzed for the presence of oxidized proteins by oxyblot analysis. The membrane was re-probed with an antibody to β-actin (n = 4). **D.** Densitometric analysis of band intensity (Image J, NIH) of oxyblot showing significant decrease in carbonyl protein groups in UA2 treated group, ** P<0.01, * P<0.05.

**Figure 7:**
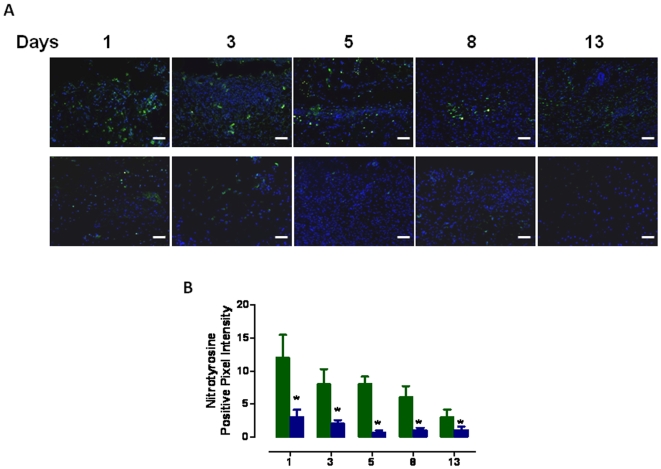
Eight wound halves, control vs. UA2 treated were analyzed by immunofluorescence for the presence of nitrotyrosine positive cells in indicated number of days post-injury. **A.** Representative images of staining. Nitrotyrosine-positive cells are green in color. Nuclei were counterstained with Hoechst 33342. Scale ba r = 50 µm. **B.** Graph showing the quantification of immunostaining. The percentage of nitrotyrosine positive cells was determined using the IP lab software (BD Biosciences). Statistical analysis was performed two- way ANOVA using GraphPad Prism version 5.00. D. (*P<0.001).

Elevated levels of oxidative stress in cells within and surrounding the damaged tissue in a wound are believed to impair the wound healing process [10] which may result, in part, from depletion of antioxidant enzymes [11]. We therefore measured relative levels of Cu/Zn-superoxide dismutase (SOD1) in dermal wound tissue from control and UA2-treated mice. SOD1 levels were two-fold greater in the wound tissue of UA2-treated mice on all post-injury days examined (days, 1, 3, 5, 8 and 13) (Figs. 6, 7). In addition to SOD-1 levels, to measure severity of oxidative stress at the wound site, we also performed oxyblot analysis with protein lysates from wound tissue from control and UA2-treated mice to determine the levels of oxidized proteins, which are characterized by the presence of carbonyl groups. In four independent experiments with different wound lysates, at days 1, 3, 5, 8 and 13, wound samples from control mice had a consistently higher protein carbonyl content compared with samples from UA2-treated mice (Fig. 6C, D). As another indicator of oxidative damage, we immunostained wound tissue sections with an antibody against nitrotyrosine, which reflects interaction of proteins with peroxinitrite, a toxic reactive oxygen species formed by the interaction of nitric oxide with superoxide anion radicals. The analysis showed that there were significantly more nitrotyrosine immunoreactive cells in the wounds of mice in the control group compared to those in the UA2-treated group (Fig. 7A, B).

## Discussion

Impaired wound healing continues to be a major health problem that predisposes to infections, long-term morbidity and mortality, particularly in high risk patients including those with diabetes or suppressed immune function, and the elderly [32]–[34]. In a previous study we screened a panel of soluble UA analogs to establish their antioxidant and neuroprotective properties and identified 1, 7 dimethyl-uric acid (UA1) and 6, 8 dithio-uric acid (UA2) as being the most effective [28]. In the present study we found that UA2 was much more effective than UA or UA1 in promoting the proliferation and migration of dermal fibroblasts, keratinocytes and vascular endothelial cells. We then tested the therapeutic potential of UA2 in a mouse model of wound healing and found that, indeed, topical application of UA greatly accelerated wound healing and enhanced the restoration of normal dermal and epidermal tissue structure in the wound area. These findings suggest a potential use of UA2 or related UA analogs in the treatment of wounds humans.

Previous findings have suggested a potential for the use of antioxidants to treat wounds. For example, Serarslan et al. [35] reported that caffeic acid phenethyl ester reduces oxidative stress and accelerates cutaneous wound healing in a rat model, and Alleva et al. [36] reported that dietary supplementation with alpha-lipoic acid enhanced wound healing in human subjects undergoing hyperbaric oxygen treatment. In addition, overexpression of manganese SOD enhanced wound healing in diabetic mice [37] and a recent study showed that application of a wound dressing with curcumin-loaded nanofibers enhanced diabetic wound healing [38]. The free radical-scavenging property of UA2 likely contributes to its beneficial effects in the in vivo and cell culture models of wound healing. Indeed, we found that levels of protein carbonyls and nitrated proteins were significantly lower in wounded tissue from UA2-treated mice compared to wounded tissue from vehicle-treated control mice. UA and some UA analogs, including UA2, have been shown to scavenge free radicals including hydroxyl radical and peroxynitrite [22], [23], [28]. Consistent with this mechanism, we previously found that UA2 was more effective than UA or UA1 in scavenging free radicals [28]. However, in the latter study UA1 and UA2 were similarly effective in reducing brain damage and improving functional outcome in a mouse model of stroke. It will therefore be of interest to evaluate the efficacy of a range of doses of UA and more soluble UA analogs in wound healing models.

We found that UA2 enhanced the proliferation and migration of vascular endothelial cells, and also accelerated the formation of vessel-like tubes by endothelial cells grown in a three-dimensional matrix. The importance of angiogenesis in wound healing [29] and our findings in the present study, suggest that similar actions of UA2 on vascular endothelial cells play an important role in the accelerated and cytoarchitecturally superior healing of full-thickness wounds treated with UA2. It remains to be determined whether antioxidant actions of UA2 account for its ability to enhance angiogenesis, or whether UA2 has other biological activities that enhance vessel formation by endothelial cells. It will be of considerable interest to determine whether UA2 might also have beneficial effects in other clinical settings where angiogenesis is impaired.

## Materials and Methods

### Uric acid analogues

In a previous study (28) we described the process for the synthesis of water soluble uric acid analogues, and characterization and preclinical development of several different UA analogs including dtUA (6, 8-dithiouricacid). In vitro and cell culture screening showed that this dtUA has high a antioxidant activity and is cytoprotective in cell culture and in vivo.

### Cell Cultures

#### Keratinocytes

Human keratinocyte cells were obtained from ATCC (# CRL-2309™) and grown in keratinocyte complete growth medium containing 0.05 mg/ml bovine pituitary extract (BPE) and 5 ng/ml epidermal growth factor (EGF) (GIBCO, Invitrogen USA). Cells were grown as a monolayer.

#### Human Micro Vascular Endothelial Cells (HMEC-1)

Human microvascular endothelial cells (HMEC-1) are Simian vacuolating virus 40 Tag (SV40 LT) transformed stable cell line provided by Dr. Fransisco Candal (Center for Disease Control, Atlanta, GA), were maintained in MCDB 131 formula (GIBCO, Invitrogen, San Diego, CA) supplemented with 10% fetal bovine serum (FBS), epidermal growth factor (EGF, 10 ng/mL), hydrocortisone (1 µg/mL), and L-glutamine (10 mmol/L).

#### Primary culture of fibroblasts

Dermal explants from the skin of young adult mice were used to harvest fibroblasts. Dermal tissue specimens were cut into ∼5 mm pieces. These fragments were placed on the surface of 100 mm Petri dishes for 40–50 minutes to allow adherence of the tissue to the culture surface. 10 ml of DMEM with 20% fetal bovine serum, penicillin (100 UI/ml) and streptomycin (100 µg/ml) (pH 7.6), at 37°C, was gently added to the culture dishes.

Cultures were maintained in a humidified incubator at 37°C in a 5% CO2/95% air atmosphere.

Cultures were passaged on reaching 80% confluence, using 0.05% trypsin/EDTA (GIBCO, Invitrogen) and the media was changed every two days, for this rate enables the maintenance of ideal conditions of pH between 7.6 and 7.8 without non-physiologic upheavals. Cells were used at passage 4 or 5 for cell migration or proliferation assays in order to minimize the influence of genetic alterations and senescent changes in the cellular morphology.

### Full-thickness wounds and quantification of healing

These methods were similar to those described previously [4]. All experiments were performed using 3–4 month-old male C57BL/6 mice. Mice were anesthetized using 2 to 2.5% vaporized inhaled isoflurane and the dorsal skin was cleansed with Betadine. Two full-thickness wounds were created in the skin on the back of each mouse using a 4 mm diameter biopsy punch (Miltex Instrument, York, PA, USA) and a biotome (Acu Punch, Acuderm Inc., Fort Lauderdale, FL, USA). Mice were treated with vehicle (10 µl of dimethylsulfoxide) or 100 µM of either UA, UA2 or UA2 applied directly to the wound site once daily in a blinded manner. Some mice in each group were euthanized on days 1, 3, 5, 8 and 13 post wounding, and skin tissue samples from the wound site were collected from all of the mice for histological and biochemical analyses. Some mice from each genotype/treatment group (n = 6–8) were evaluated daily for 13 days following wounding. Digital photographs of the injury site were taken with a standard-sized dot placed beside the wound; wound size was expressed as the ratio of the wound area to the dot measurement.

### Measurement of wound healing rate

Measurements of length and width were done using a caliper. The first post-incision wound measurement was made on day 0. The measurements were done without knowledge of the treatment history of the mice. Wound area was calculated using digital planimetry. Linear healing progress (LHP) was determined using the following formula [39]

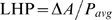
where, DA represents change in wound area between first and last days of healing period and P_avg_ stands for the mean wound perimeter at the same days:
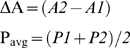



Linear healing rate (LHR, mm/day) was measured using the next equation:
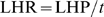
where, t represents the healing period in days [39].

### Tissue preparation and examination

The biopsy specimens involving the central part of the wounds (Days 1, 3 5, 8 and 13) were obtained perpendicularly to the dorsal midline from mice for light microscopy. Skin specimens were fixed in formalin, dehydrated through a graded series of ethanols, cleared in xylene, and embedded in paraffin wax. 5 µm thick sections were prepared and stained with hematoxylin-eosin. The histomorphometric method was an adaptation of the point-counting procedure. The counting procedure in each section was performed at a total magnification of 200 in 3 random fields per section limited to the wounded area. Two components of each section were examined in specimens associated with 4 mice per time interval: mononuclear inflammatory cells (MICs) and blood vessels on days 5. After transferring the images to the computer, a 252-square graticule was superimposed on the screen over the wounded site to facilitate counting. The epithelium was assessed on days 5 standard histologic grading system.

### Histology

To assess cellular infiltration into the wounded area, samples from three mice per group were collected on days 1, 3, 5, 8 and 13 during the healing process. To obtain skin samples from the biopsied areas, mice were euthanized with an overdose of sodium pentobarbital and the tissues were subsequently removed by dissection. Formalin-fixed samples were sectioned at 4 µm and stained with hematoxylin and eosin. All the slides were evaluated by two veterinary pathologists (S. C. and S. P.) in blinded manner.

### Immunoblot analysis

Tissue protein was extracted using T-PER tissue protein extraction buffer with protease inhibitor cocktail (Sigma). Methods for protein quantitation, electrophoretic separation, and transfer to nitrocellulose membranes were as described previously [40]. Membranes were incubated in blocking solution (5% milk in Tween Tris-buffered saline; TTBS) overnight at 4°C followed by a 1 h incubation in primary antibody diluted in blocking solution at room temperature. Membranes were then incubated for 1 h in secondary antibody conjugated to horseradish peroxidase and bands were visualized using a chemiluminiscence detection kit (ECL, Amersham). The primary antibodies were. SOD1 (Abcam, Cambridge, MA USA) and an actin antibody (Sigma St. Louis, MO USA).

### Detection of Protein Carbonyls by Oxyblot

Protein carbonyls in tissue protein were assayed with a protein oxidation detection kit (Oxyblot; Cell Biolabs, San Diego, CA). The tissue protein samples were prepared for electrophoresis with 4X reducing SDS sample buffer. The gel proteins were transferred to a PVDF membrane. The membrane was immersed in 100% methanol and dried at room temperature and then equilibrated with TBS containing 20% methanol. After washing with 2 N HCl the derivatization of the carbonyl groups of proteins by Dinitrophenyl hydrazine (DNPH) was performed on 20 µg of tissue proteins for 5 minutes at room temperature. The reaction was stopped with 2 N HCl and the membrane was washed two times with 100% methanol. The blocking was one hour with 5% non fat dry milk in TBST and incubated with Rabbit anti-DNP antibody (1∶1000) at 4°C overnight. The membrane was washed three times and then incubated with secondary antibody goat anti-rabbit IgG, HRP-conjugate (1∶3000) for one hour at room temperature and then washed with TBST. The oxidized proteins were detected by chemiluminescence (ECL Thermoscientific).

### Immunofluorescence

4-mm punch biopsy wounds, including the wound edge, were harvested on days 1, 3, 5, 8, and 13 from control and UA2 treated mice. Skin tissue was embedded in Optimal Cutting Temperature (OCT) compound and frozen. Sections (6 µm) were cut with a cryostat and fixed in acetone. Subsequently sections were blocked with 10% goat serum before being incubated with rabbit anti- alpha smooth muscle actin (1∶200; Abcam) and mouse anti- nitrotyrosine (1∶200; Zymed) overnight at 4°C. After being washed, the sections were incubated in anti-rabbit and anti mouse IgG conjugated to Alexa 568 and 488 respectively for 45 min at room temperature (both 1∶200). Sections were counterstained with Hoechst 33342 (Invitrogen) visualized under a Nikon Eclipse 80*i* microscope.

### Quantification of immunohistochemistry

Using spatially calibrated images with the automated measurement tools in IP lab software (BD Biosciences Bio-imaging, Rockville, MD) total area of positive pixel intensity was measured and analyzed with two-way ANOVA using GraphPad Prism version 5.00 for Windows, GraphPad Software, San Diego California USA.

### Endothelial cell scratch wound healing assay

Human Microvascular Endothelial Cells (HMEC-1 cells), human keratinocytes and mouse fibroblasts were seeded into 60 mm plates and grown to confluency. After 24 hours of serum starvation (DMEM supplemented with 1% FBS), cells were treated with either vehicle, UA, UA1 or UA2 (15 µM). The cell monolayer was then subjected to a mechanical scratch-wound induced using a sterile pipette tip. Cells were then cultured for additional period of 24 hours in a serum-free basal medium in the continued presence of vehicle, UA, UA1 or UA2. Cells were then fixed in a solution of 4% paraformaldehyde in PBS and stained with crystal violet. Cells in the injury area were visualized under phase-contrast optics (10X objective) and the number of cells which had migrated into the initially cell-free scratch area was counted.

### Endothelial tube formation and chemotaxis cell migration assays

HMEC-1 cells (1×10^3^ cells/well) were dispensed to Matrigel-coated 8-well chamber slides (Lab-Tek, Nalge Nunc International, Rochester, NY, USA) in 125 µl of EGM-2 containing either vehicle, UA, UA1 or UA2 (15 µM) and incubated for 18 hours. The cells were then visualized by microscopy and tube formation was scored as described previously [4], [41]. Cell migration analysis was performed using Transwell membrane filters (Corning, Costar) containing a polycarbonate filter with 8 µm pores. The bottom chamber was filled with complete growth medium containing chemoattractant growth factors. Cells (5×10^4^ in 100 µl) were seeded into each transwell with EGM containing 0.2% fetal bovine serum with vehicle, UA, UA1 or UA2 (15 µM) and allowed to migrate for 6 hours. At the end of the incubation, non-migrated cells remaining in the transwell insert were removed. The migrated cells (on the outer bottom of the transwell) were fixed with methanol and stained with hematoxylin and eosin, and the stained cells were counted in 5 or more random 100X fields. Each experiment was performed in triplicate, and the experiment was repeated twice. Growth correction was not applied because no increase in the cell number was observed during the incubation period of 6 hours.

### Quantification of cell proliferation

The proliferation of cultured endothelial cells, keratinocytes and fibroblasts was measured using a colorimetric assay. Cells (1×10^4^) were incubated with either vehicle, UA, UA1 or UA2 (15 µM) for 24, 48 and 72 hours. Then 10 µl of 3-(4, 5dimethylthiazol-2-yl)-2,5-diphenyl-2*H*-tetrazolium bromide (MTT) solution (R&D Systems Inc. Minneapolis, MN) was added to each well and the cells were incubated for a further 4 hours at 37°C. After the cells were washed 3 times with PBS (pH 7.4), the insoluble formazan product was dissolved by incubation with 100 µl detergent for 2 hours. The absorbance of each well was measured on an enzyme-linked immunosorbent assay (ELISA) micro-plate reader at 570 nm. Each experiment was performed in quadruplicate. The proliferation rate was calculated as follows: (absorbance experimental/Absorbance control-1) ×100.

## Supporting Information

File S1 (0.02 MB RTF)
