# Supplementary material for: A Synthetic Uric Acid Analog Accelerates Cutaneous Wound Healing in Mice
Source: PLoS One. 2010 Apr 6;5(4):e10044. doi: 10.1371/journal.pone.0010044 (PMC2850366; doi:10.1371/journal.pone.0010044)
Supplement: File S1 — (0.02 MB RTF) [file pone.0010044.s001.rtf]

Supplemental Information: Histological Evaluation (these descriptions are based on the evaluation of 12 wounds in 6 mice for each treatment group)
Control Mice 
Day 1: Polymorphonuclear and mononuclear cells are present at the wound area. Edema is present with expanded space in the deep dermis and the near wound site.  The skin edges are healthy and the wound extends to the surface of the deep dermis.  The superficial layer of the wound is lined by a thin crusting.   Edema and fibrin strands are evident, and accumulations of mononuclear and polymorphonuclear cells are present. 60X (Insert): Fibrin strands with mononuclear and polymorphonuclear cells are present. 
Day 3: Edema and fibrous appearance have resolved significantly. The interstitial layer and the dermis have greater numbers of mononuclear cells. The wound edge with adjacent healthy skin is clearly present. 60X (Insert): Neovasulature with erythrocytes and abundant mononuclear cells are visible. 
Day 5: The edema and fibrin has considerably disappeared in contrast to day 3. The collagen fibers are unsettled in the midst of numerous fibroblasts, and new small capillaries have begun to appear. Increased numbers of fibroblasts and neo-vascularization are evident There is a moderately attached eschar on the surface of the wound with a divergent demarcation between the healthy and wound edges. 60X (Insert): New blood vessels with fibrobalsts are present.
Day 8: The wound show a distinctly tapered appearance, an indication of wound closure. There is discrete constriction of the wound due to scar tissue formation, typified by numerous new blood vessels, fibroblasts and moderately organized collagen.  More organized connective tissue and neo-vascularization are evident. 60X (Insert): Moderately organized collagen with blood vessels containing erythrocytes is present along with fibroblasts. 
Day 13: The wound has been contracted and the dermis is very well organized in comparison to day 8. The collagen fibers are thick with a predisposition to form irregular bundles. The wound site dermis has less dense collagen compared to the adjacent healthy dermis.    There is a demarcation of healthy and wound site within the dermis.  The staining of the healthy side is more distinctly pink due to well organized bundles of collagen. 60X (Insert): Irregularly organized collagen with new blood vessels is present.

UA2 treated mice 
Day 1:  The interstitial space of the deep dermis is edematous and has necrotic cell debris. The wound has widespread crusting and the edges are clearly demarcated. There is a prevalence of fibrin, mononuclear and polymorphonuclear cells.  The wound extended to the surface of the deep dermis. 60X (Insert): Abundant mononuclear and polymorhonuclear cells along with erythrocytes are seen. 
Day 3: Fibrin and cell infiltration is modest. Inflammatory phase is still the most predominant feature but less edematous than in control mice. Fibroblasts and new blood vessels are evident. Widespread crusting is still prominent. The keratinocyte layer has distinct desmosomal junctions. 60X (Insert): Mononuclear and polymorhonuclear cells along with fibroblasts are present.
Day 5: Proliferation of several cell types giving the tissue a densely populated appearance. Separate demarcation of the wound edges and fibrin is absent in the wound site. The amount of collagen is relatively low compared with the enhanced numbers of cells. There are numerous fibroblasts and fewer mononuclear cells with poorly organized collagen fibers. 60X (Insert): proliferation of several cellular types along with neovasculature is present. 
Day 8:  The wound has a low amount of mature collagen. Clearly demarcated wound edges, with the healthy edge having distinctly mature collagen along with associated adnexal structures and a thin hyperkeratotic keratinocyte layer is evident.  Contracted scar tissue and the deeper layer have infiltration of adipocytes. The wound appears closed and healed, but is distinct from adjacent undamaged skin. 60X (Insert): Fairly well organized collagen fibers are present.

Day 13: The wound is completely healed.  Keratinocyte layer, fibroblasts and endothelial cells formed an integral part of the repaired tissue.  Collagen at the wound site is very well organized. 60X (Insert): Keratinocytes, fibroblasts and endothelial cells aare well organized; collagen organization is almost comparable to adjacent unwounded site. Scale bar for all the inserts represent 15ìm. 
